# Supplementary material for: Neutrophil-induced ferroptosis promotes tumor necrosis in glioblastoma progression
Source: Nat Commun. 2020 Oct 27;11:5424. doi: 10.1038/s41467-020-19193-y (PMC7591536; doi:10.1038/s41467-020-19193-y)
Supplement: Supplementary file 1 — Supplementary Information [file 41467_2020_19193_MOESM1_ESM.pdf]

Supplementary Information

Neutrophil-induced ferroptosis promotes tumor necrosis in glioblastoma progression

Patricia P. Yee[1, 2], Yiju Wei[1], Soo-Yeon Kim[1], Tong Lu[1], Stephen Y. Chih[2], Cynthia Lawson[3], Miaolu Tang[1], Zhijun Liu[1], Benjamin Anderson[1], Krishnamoorthy Thamburaj[4], Megan M. Young[1, 5], Dawit G. Aregawi[6, 7, 8], Michael J. Glantz[6, 7, 8], Brad E. Zacharia[6, 7, 9], Charles S. Specht[3, 6, 10], Hong-Gang Wang[1, 5], and Wei Li[1, 11 *]

Affiliations

[1]Division of Hematology and Oncology, Department of Pediatrics, Penn State College of Medicine, Hershey, PA, USA

[2]Medical Scientist Training Program, Penn State College of Medicine, Hershey, PA, USA

[3]Division of Anatomic Pathology, Department of Pathology and Laboratory Medicine, Penn State College of Medicine, Hershey, PA, USA

[4]Department of Radiology, Penn State College of Medicine, Hershey, PA, USA

[5]Department of Pharmacology, Penn State College of Medicine, Hershey, PA, USA

[6]Division of Neuro-Oncology and Skull Base Surgery, Department of Neurosurgery, Penn State College of Medicine, Hershey, PA, USA

[7]Penn State Cancer Institute, Penn State College of Medicine, Hershey, PA, USA

[8]Department of Medicine, Penn State College of Medicine, Hershey, PA, USA

[9]Department of Otolaryngology-Head and Neck Surgery, Penn State College of Medicine, Hershey, PA, USA

[10]Department of Neurology, Penn State College of Medicine, Hershey, PA, USA

[11]Department of Biochemistry and Molecular Biology, Penn State College of Medicine, Hershey, PA, USA

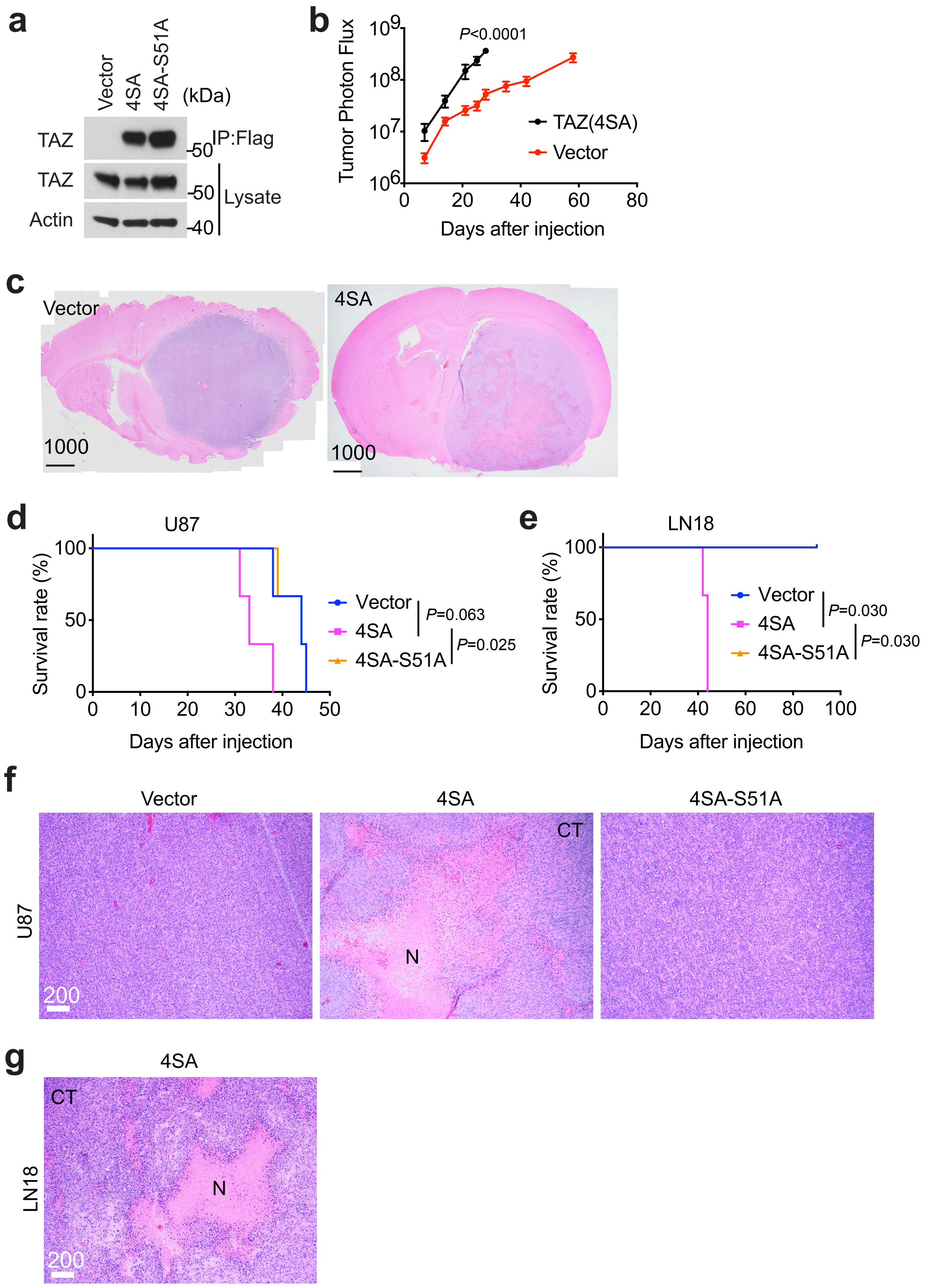

Supplementary Figure 1

Supplementary Figure 1. Related to Figure 1. (a) Flag-TAZ$^{(4SA)}$, Flag-TAZ$^{(4SA-S51A)}$ or vector-transduced LN229 cells were subjected to Flag-mediated immunoprecipitation followed by western blotting as indicated. **(b)** Tumor photon flux from tumor bioluminescence imaging (BLI) of mice implanted with LN229$^{TAZ(4SA)}$ or LN229^{vector} cells (*n* = 5 tumors for each group). Data are presented as mean ± s.e.m. Unpaired two-tailed *t*-test. **(c)** Representative H&E-stained formaldehyde-fixed paraffin-embedded brain sections obtained from mice implanted with LN229^{vector} (left) and LN229$^{TAZ(4SA)}$ (right) upon reaching same tumor burden as measured by tumor BLI (*n* = 3 tumors for each group). **(d), (e)** Kaplan-Meier survival curves of mice implanted with U87 (f) or LN18 (g) stably expressing empty vector control (*n* = 3 mice), TAZ^{4SA} (*n* = 3 mice), or TAZ$^{4SA-S51A}$ (*n* = 3 mice). Mice implanted with LN18^{vector} or LN18$^{TAZ(4SA-S51A)}$ do not develop tumors. Two-sided log-rank test. **(f), (g)** Representative H&E-stained formaldehyde-fixed paraffin-embedded sections of mice implanted with U87 (f) and LN18 (g) transduced with indicated genes as described above; N denotes central tumor necrosis, whereas CT denotes cellular tumor (*n* = 3 mice for each group). All scale bars are in μm. Source data are provided as a Source Data file.

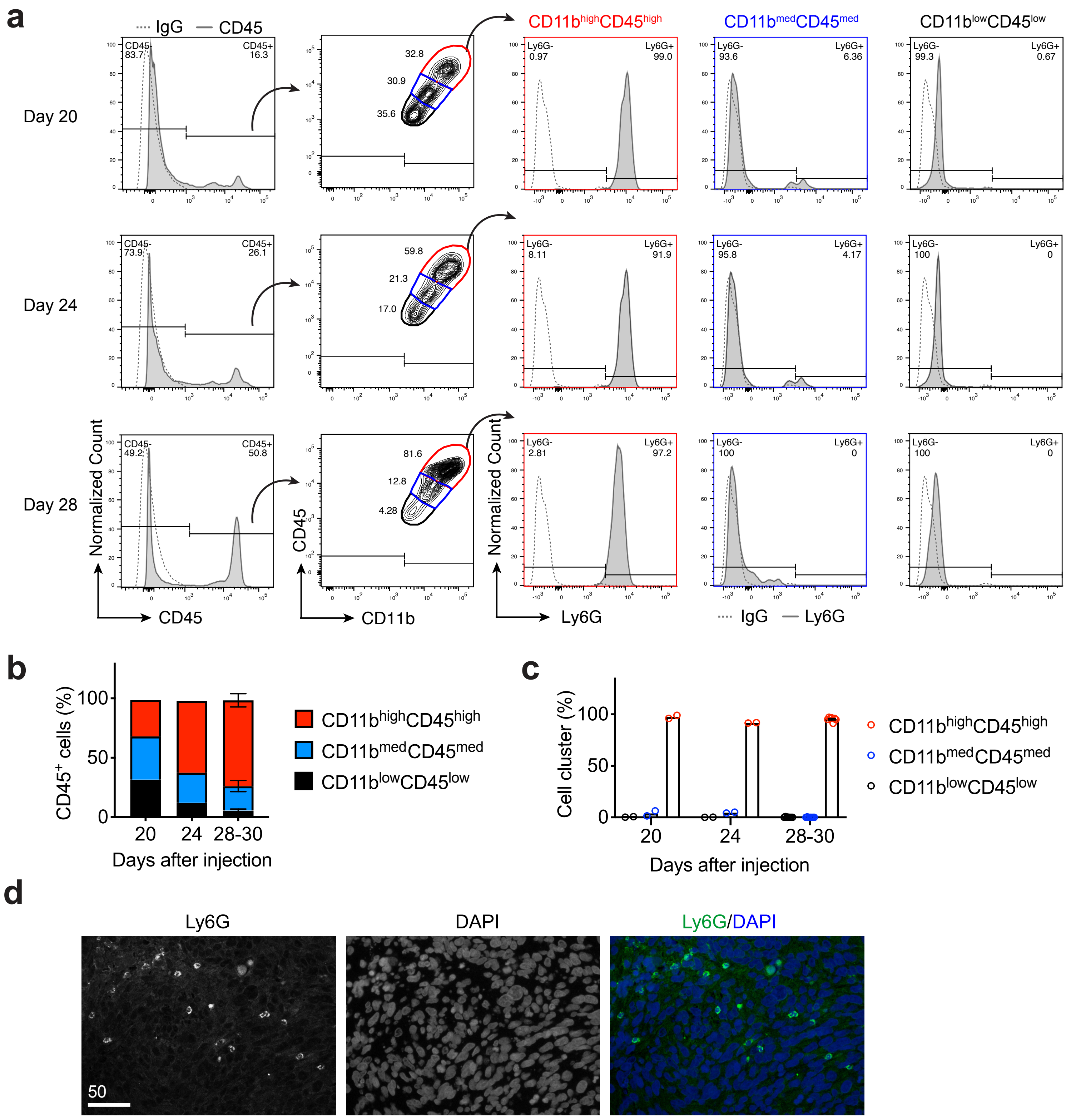

Supplementary Figure 2

Supplementary Figure 2. Related to Figure 2. (a) Representative flow cytometry analyses of murine myeloid cell markers, CD11b and CD45, and murine neutrophil marker, Ly6G, on cells isolated from tumor tissues of mice implanted with $LN229^{TAZ(4SA)}$ on days 20, 24, and 28 (i.e. endpoints) after tumor implantation (one animal is shown for each time point). Tumor cells were first excluded as $CD45^{-}$ cells (first column), followed by contour plotting of CD11b and CD45 (second column). Three major populations were visualized based on contour dot plots, and they were separated into $CD11b^{high}CD45^{high}$ (red), $CD11b^{med}CD45^{med}$ (blue), and $CD11b^{low}CD45^{low}$ (black) populations based on respective CD11b and CD45 signal intensities. Following such separation, the percentage of neutrophils in each population was examined via Ly6G flow cytometry (shown in the third column for $CD11b^{high}CD45^{high}$, fourth column for $CD11b^{med}CD45^{med}$, and fifth column for $CD11b^{low}CD45^{low}$ cell clusters). **(b)** Percentages of $CD11b^{high}CD45^{high}$ (red), $CD11b^{med}CD45^{med}$ (blue), and $CD11b^{low}CD45^{low}$ (black) cells among $CD45^{+}$ cells within tumor tissues collected from mice implanted with $LN229^{TAZ(4SA)}$ cells on days 20 (n = 2 tumors), 24 (n = 2 tumors), and 28-30 (i.e. endpoints; n = 7 tumors) after tumor implantation. Data are presented as mean ± s.e.m. **(c)** Percentages of neutrophils (i.e. $Ly6G^{+}$ cells) among $CD11b^{high}CD45^{high}$ (red circles), $CD11b^{med}CD45^{med}$ (blue circles), and $CD11b^{low}CD45^{low}$ (black circles) cell clusters within tumor tissues collected from mice implanted with $LN229^{TAZ(4SA)}$ cells on days 20 (n = 2 tumors), 24 (n = 2 tumors), and 28 (endpoints; n = 7 tumors) after tumor implantation. Data are presented as mean ± s.e.m. Each data point represents an animal. **(d)** Representative immunofluorescent staining of mouse-specific neutrophil marker, Ly6G, and DAPI staining on paraffin-embedded section of mouse implanted with $LN229^{TAZ(4SA)}$ collected 16 days after tumor implantation (n = 3 tumors). This section was obtained from a cut adjacent to the one shown in the bottom of Figure 2g. All scale bars are in μm. Source data are provided as a Source Data file.

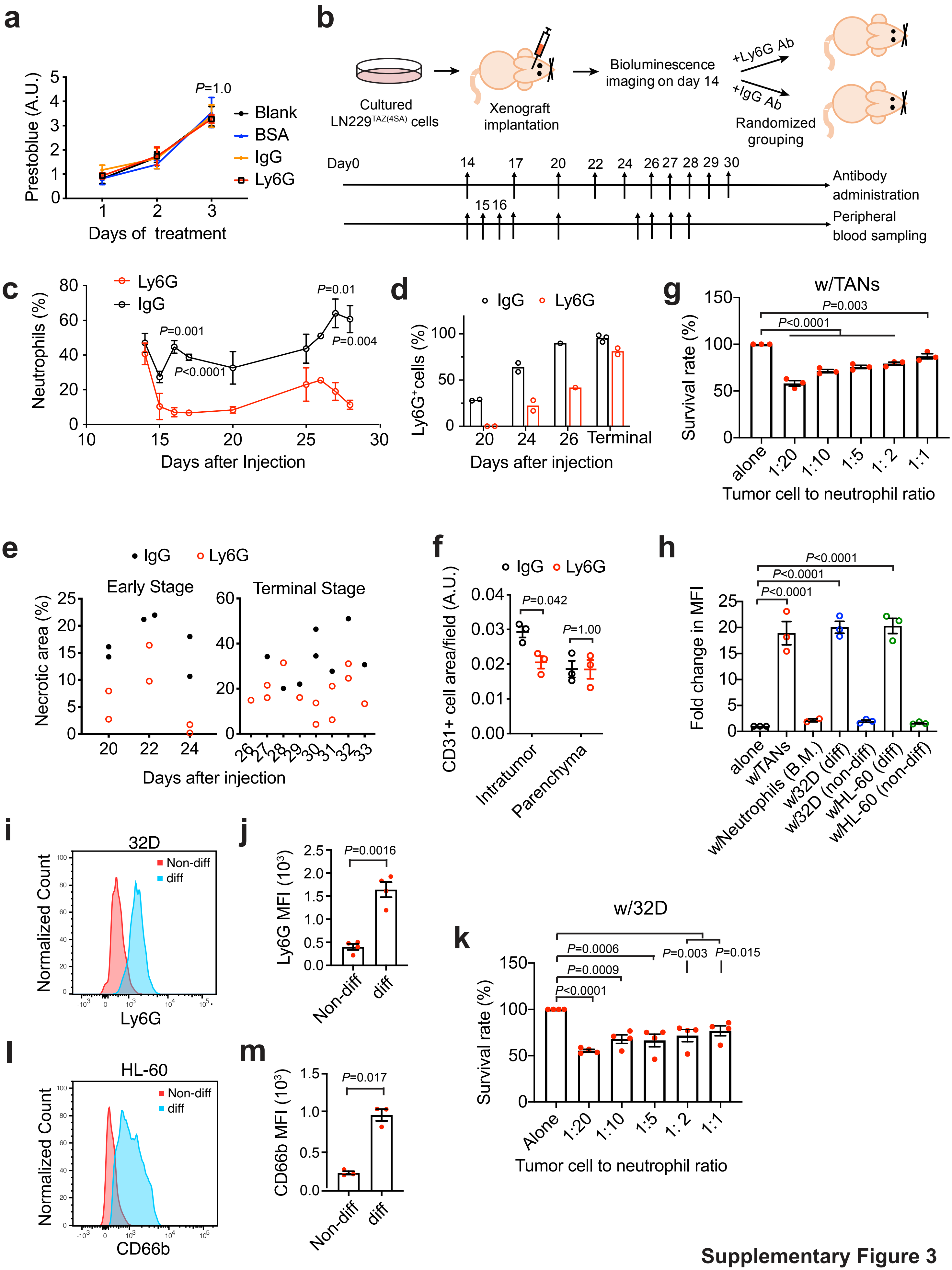

Supplementary Figure 3

Supplementary Figure 3. Related to Figure 3. (a) LN229$^{TAZ(4SA)}$ cell proliferation after 1-3-day treatments with bovine serum albumin (BSA), IgG, or Ly6G quantified by PrestoBlue fluorescence. *P*-value of Day 3 is indicated. One-way ANOVA. *n* = 3 independent experiments for each treatment. **(b)** Schematic illustrating neutrophil depletion via Ly6G. **(c)** Neutrophils (as % of WBCs) in blood smears of Ly6G- or IgG-treated mice; *n* = 3 mice for each treatment at each time, except for day 26 (n_{IgG} = 1 mouse, n_{Ly6G} = 2 mice). Unpaired two-tailed *t*-test. **(d)** Ly6G^{+} cells as percentage of CD11b^{+}CD45^{+} cells from LN229$^{TAZ(4SA)}$ tumors treated as indicated. For each treatment, day 20 and 24: *n* = 2 mice; day 26: *n* = 1 mouse; terminal: *n* = 3 (IgG) or 2 mice (Ly6G). **(e)** Values in Figure 3a plotted by day of tissue collection. Each point represents an animal treated as indicated. **(f)** Total CD31^{+} cell area/field within and outside tumors from IgG- or Ly6G-treated mice at endpoints (*n* = 3 tumors each). Unpaired two-tailed *t*-test. **(g)** Viability (luminescence) of LN229$^{TAZ(4SA)}$ cells cultured alone or with TANs at various tumor-to-neutrophil ratios. Survival normalized to LN229$^{TAZ(4SA)}$ cells cultured alone. One-way ANOVA. *n* = 3 independent experiments each. **(h)** Viability (Sytox-Green median fluorescence intensity, MFI) of LN229$^{TAZ(4SA)}$ cells cultured alone or with indicated neutrophils. Non-diff, undifferentiated; diff, differentiated; B.M., bone marrow. One-way ANOVA. *n* = 3 independent experiments each. **(i), (j)** Flow cytometry of Ly6G (i) and quantification of Ly6G-MFI (j) on undifferentiated and differentiated 32D cells. Paired two-tailed *t*-test. *n* = 4 independent experiments each. **(k)** Viability (luminescence) of LN229$^{TAZ(4SA)}$ cells cultured alone or with differentiated 32D cells at various tumor-to-neutrophil ratios. Survival normalized to LN229$^{TAZ(4SA)}$ cells cultured alone. One-way ANOVA. *n* = 4 independent experiments each. **(l), (m)** Flow cytometry of CD66b (l) and quantification of CD66b-MFI (m) on undifferentiated and differentiated HL-60 cells. Paired two-tailed *t*-test. *n* = 3 independent experiments each. **(a-m)** Data are presented as mean ± s.e.m. Source data are provided as a Source Data file.

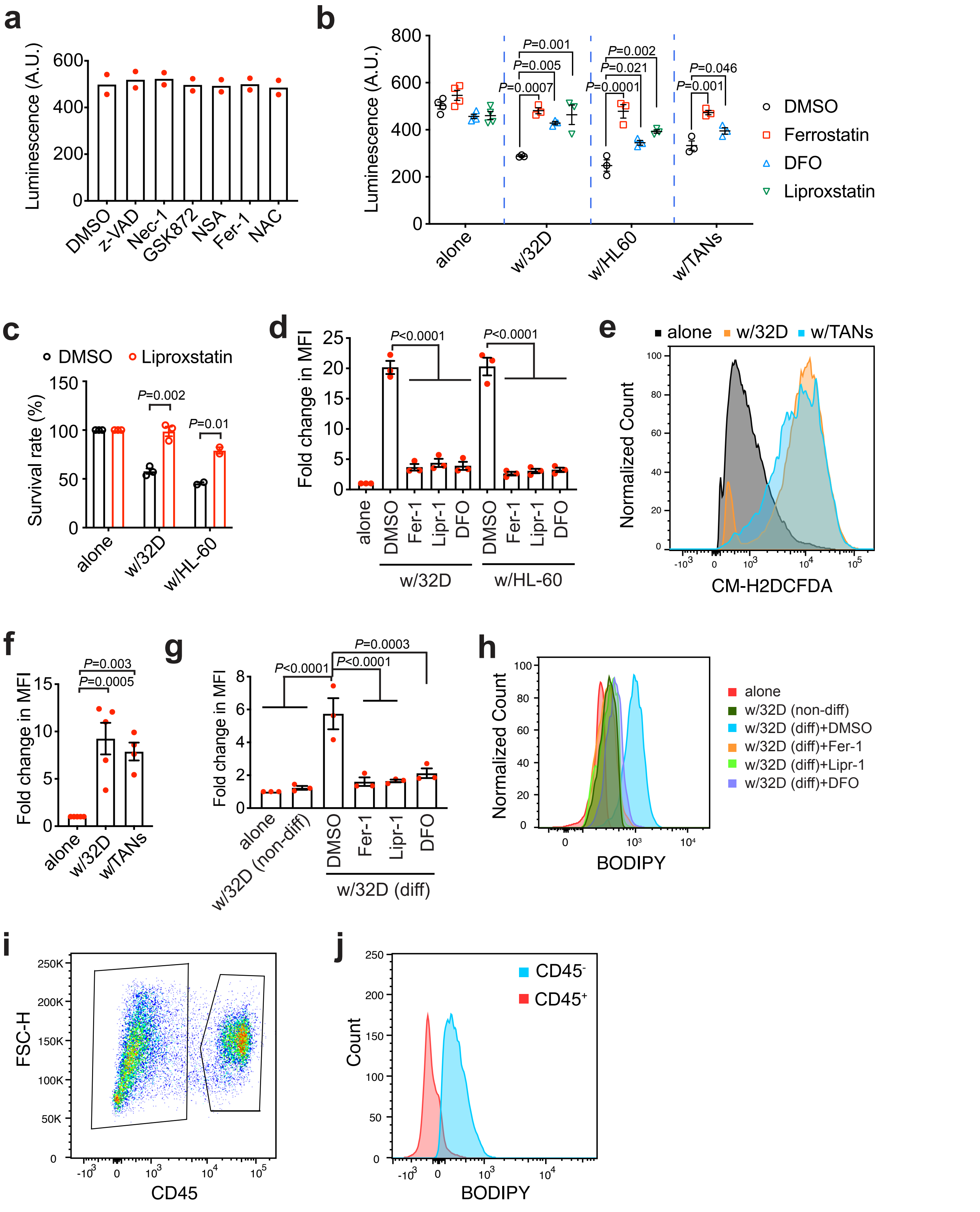

Supplementary Figure 4

Supplementary Figure 4. Related to Figure 4. (a) Viability (luminescence) of $LN229^{TAZ(4SA)}$ cells cultured alone and treated with or without each inhibitor tested in Figure 4a. **(b)** Viability (luminescence) of $LN229^{TAZ(4SA)}$ cells cultured alone, with differentiated 32D or HL-60 cells, or with $LN229^{TAZ(4SA)}$-TANs and treated with 2 µM ferrostatin-1, 0.1 (w/32D) or 0.2 (w/HL-60 or TANs) mM deferoxamine (DFO), 0.2 µM liproxstatin-1, or DMSO. One-way ANOVA. **(c)** Viability (luminescence) of $LN229^{TAZ(4SA)}$ cells cultured alone or with differentiated 32D or HL-60 cells and treated with 0.2 µM liproxstatin-1 or DMSO. Survival normalized to $LN229^{TAZ(4SA)}$ cells cultured alone and treated with the same compound. Unpaired two-tailed *t*-test. **(d)** Viability (by Sytox-Green MFI) of $LN229^{TAZ(4SA)}$ cells cultured alone or with differentiated 32D or HL-60 cells and treated with either 2 µM ferrostatin-1 (Fer-1), 0.1 (w/32D) or 0.2 (w/HL-60 or TANs) mM deferoxamine (DFO), 0.2 µM liproxstatin-1 (Lipr-1), or DMSO. One-way ANOVA. **(e)** Representative flow cytometry of ROS-sensing CM-H2DCFDA reagent in $LN229^{TAZ(4SA)}$ cells cultured alone (*n* = 5 independent experiments), with $LN229^{TAZ(4SA)}$-TANs (*n* = 4 independent experiments), or with differentiated 32D (*n* = 5 independent experiments). **(f)** Fold change in CM-H2DCFDA MFI normalized to $LN229^{TAZ(4SA)}$ cells cultured alone as in (e). One-way ANOVA. **(g), (h)** BODIPY 581/591 C11 MFI via flow cytometry on $LN229^{TAZ(4SA)}$ tumor cells cultured alone or with undifferentiated (non-diff) or differentiated (diff) 32D cells and treated with 2 µM ferrostatin-1 (Fer-1), 0.1 mM deferoxamine (DFO), 0.2 µM liproxstatin-1 (Lipr-1), or DMSO. Data plotted as fold change in MFI normalized to $LN229^{TAZ(4SA)}$ cells cultured alone and treated with the same compound (g), and representative BODIPY 581/591 flow cytometry analysis is shown in (h). One-way ANOVA. **(i), (j)** Representative flow cytometry and corresponding gating scheme of BODIPY 581/591 C11 fluorescence in $CD45^-$ tumor and $CD45^+$ murine immune cells (gated as in (i)) isolated from $LN229^{TAZ(4SA)}$ mouse brains in Figure 4h (*n* = 7 tumors). **(a-h)** Data are presented as mean ± s.e.m. Each data point represents an animal or an average of replicates from an independent experiment. Source data are provided as a Source Data file.

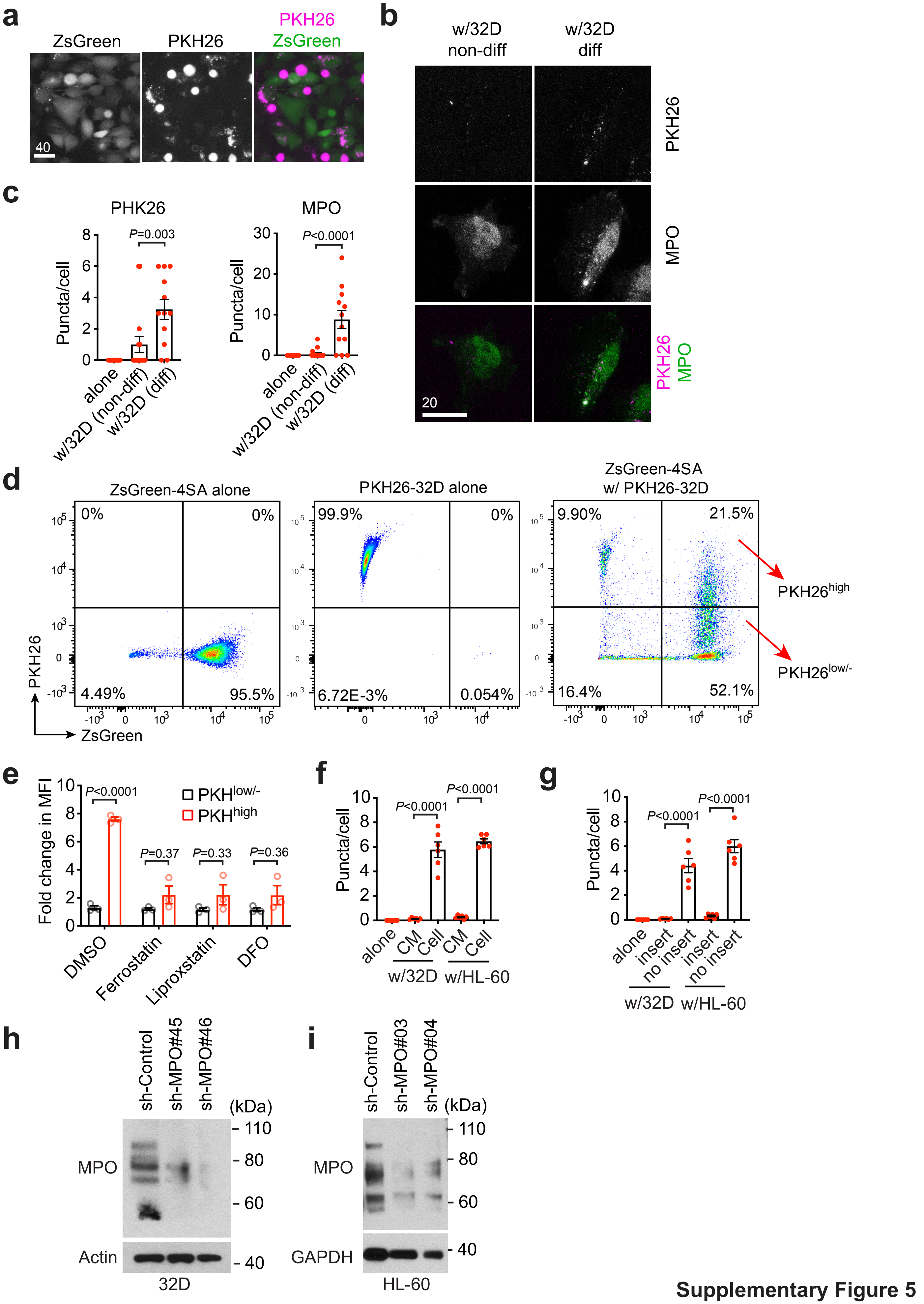

Supplementary Figure 5

Supplementary Figure 5. Related to Figure 5. (a) Images of LN229$^{TAZ(4SA)}$ cells expressing ZsGreen cocultured with PKH26-labeled differentiated 32D cells. *n* = 3 independent experiments. **(b)** MPO immunofluorescence staining of LN229$^{TAZ(4SA)}$ cells cultured with PKH26-labeled, undifferentiated (non-diff, *n* = 10 image fields) or differentiated (diff, *n* = 12 image fields) 32D cells. *n* is as in Figure 5b. **(c)** Number of PKH26 (left) and MPO (right) puncta/cell of the corresponding images in (b) and LN229$^{TAZ(4SA)}$ cells cultured alone (*n* = 16 image fields). One-way ANOVA. **(d)** Schematic of sorting of ZsGreen-expressing LN229$^{TAZ(4SA)}$ cells with many (PKH^{high}) or few (PKH$^{low/-}$) PKH26 puncta as shown in Figures 5d and 5e. **(e)** Viability (Sytox-Blue MFI) of ZsGreen-expressing LN229$^{TAZ(4SA)}$ cells with many or few intracellular PKH26 puncta when cocultured with PKH26-labeled, differentiated 32D cells and treated with 2 µM ferrostatin-1 (Fer-1), 0.1 mM deferoxamine (DFO), 0.2 µM liproxstatin-1 (Lipr-1), or DMSO. Data plotted as fold change in MFI normalized to PKH$^{low/-}$-LN229$^{TAZ(4SA)}$ cells treated with the same compound in each condition. *n* = 3 independent experiments for each condition. Unpaired two-tailed *t*-test. **(f)** Number of MPO puncta/cell of MPO immunofluorescent staining of LN229$^{TAZ(4SA)}$ cells cultured alone, with differentiated 32D or HL-60 cells (denoted as cell), or with conditioned media (denoted as CM) derived from differentiated 32D or HL-60 monoculture. *n* = 6 image fields for each condition. Unpaired two-tailed *t*-test. **(g)** Number of MPO puncta/cell with MPO immunofluorescent staining of LN229$^{TAZ(4SA)}$ cells, cultured either alone or with differentiated 32D or HL-60 cells seeded within or without the cell-impermeable 0.4 µm pore size polyethylene terephthalate Millicell hanging cell culture insert. *n* = 6 image fields for each condition. For (f) and (g), an image field contains an average of 80-100 cells; images were taken from two independent experiments. Unpaired two-tailed *t*-test. **(h), (i)** Differentiated 32D or HL-60 cells transduced with indicated shRNAs (one scrambled control and two shRNAs each) were lysed and subjected to western blotting for indicated proteins. All scale bars are in µm. **(c-g)** Numerical data are presented as mean ± s.e.m. Source data are provided as a Source Data file.

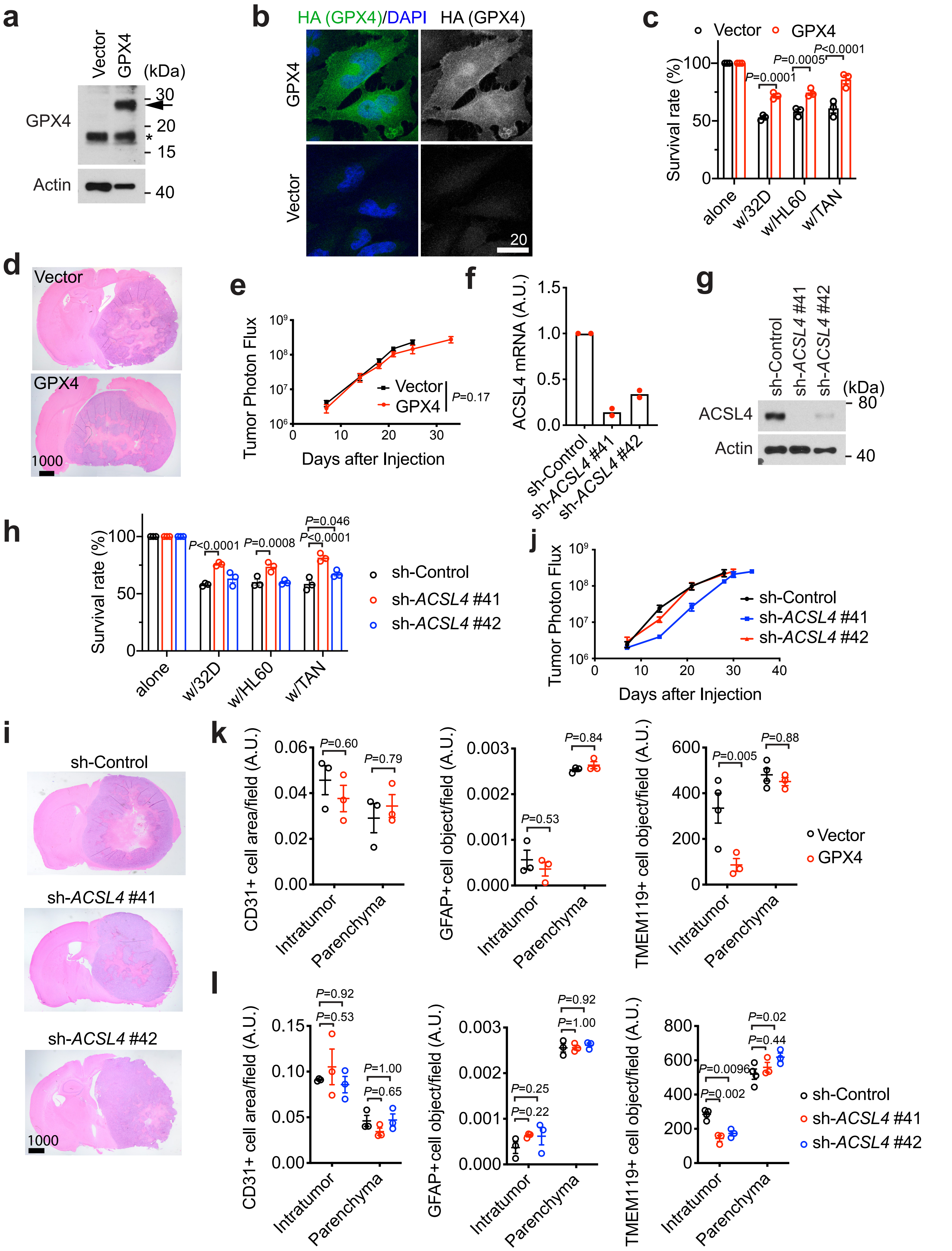

Supplementary Figure 6

Supplementary Figure 6. Related to Figure 6. (a) Western blotting of GPX4 in lysates of LN229$^{TAZ(4SA)}$ cells transduced with vector or rGPX4. Endogenous GPX4 (asterisk); rGPX4 (arrow). **(b)** Immunofluorescence staining of LN229$^{TAZ(4SA)}$ cells as in (a). rGPX4 is tagged by Flag-HA (see Methods). **(c)** Viability (luminescence) of LN229$^{TAZ(4SA)-Vector}$ and LN229$^{TAZ(4SA)-rGPX4}$ cells cultured alone, with differentiated 32D or HL-60 cells, or with LN229$^{TAZ(4SA)}$-TANs (*n* = 3 independent experiments for each group). Survival normalized to LN229$^{TAZ(4SA)-Vector}$ or LN229$^{TAZ(4SA)-rGPX4}$ cells cultured alone. Two-way ANOVA. **(d)** H&E brain sections from mice implanted with LN229$^{TAZ(4SA)}$ cells transduced with vector or rGPX4 (*n* = 5 tumors for each). **(e)** Tumor burden as measured via BLI of mice implanted with LN229$^{TAZ(4SA)}$ cells transduced as in (d). *n* = 5 tumors for each group. Unpaired two-tailed *t*-test. **(f), (g)** Verification of ACSL4 knockdown in LN229$^{TAZ(4SA)}$ cells transduced with indicated shRNAs via q-RT-PCR for *ACSL4* mRNAs (f) and via western blotting for ACSL4 proteins (g). **(h)** Viability (luminescence) of LN229$^{TAZ(4SA)}$ cells transduced with scrambled shRNA (sh-Control) or either of two shRNAs targeting *ACSL4* (#41 and #42) cultured alone, with differentiated 32D or HL-60 cells, or with LN229$^{TAZ(4SA)}$-TANs (*n* = 3 independent experiments for each group). Survival normalized to LN229$^{TAZ(4SA)}$ cells transduced with indicated shRNAs cultured alone. Two-way ANOVA. **(i)** H&E brain sections from mice implanted with LN229$^{TAZ(4SA)}$ cells transduced as in (h); sh-Control, *n* = 7 tumors; sh#41, *n* = 7 tumors; sh #42, *n* = 6 tumors. **(j)** Tumor burden as measured via BLI of mice implanted with LN229$^{TAZ(4SA)}$ cells transduced as in (h). *n* = 5 tumors for each group. **(k) and (l)** Quantification of endothelial cells, astrocytes, and microglia via CD31, GFAP, or TMEM119 immunohistochemistry on brain sections from mice implanted with tumor cells as in (d) for (k), or as in (i) for (l). *n* = 3-4 tumors for each group. Two-way ANOVA. **(b-l)** Each data point represents an animal or an average of replicates from an independent experiment. Numerical data are presented as mean $\pm$ s.e.m. Scale bars are in μm. Source data are provided as a Source Data file.

a

Positive Role

ACSL4	*PTGS2*	*HMOX1*	*ABCC1*
CARS	*RPL8*	*ACO1*	*SLC1A5*
CHAC1	*SAT1*	*STEAP3*	*GOT1*
CS	*FDFT1*	*ACSF2*	*G6PD*
DPP4	*TFRC*	*LOX*	*PGD*
GLS2	*PHKG2*	*PEBP1*	*ZEB1*
LPCAT3	*KEAP1*	*SQLE*	*ALOX12*
NCOA4	*IREB2*	*NOX1*	*ALOX15*

Negative Role

AKR1C1	*GSS*	*ACSL3*
AKR1C2	*HMGCR*	*NQO1*
AKR1C3	*HSPB1*	*CBS*
CISD1	*CRYAB*	*SLC7A11*
FANCD2	*MT1G*	
GCLC	*NFE2L2*	
GCLM	*FTH1*	
GPX4	*NFS1*	

Supplementary Figure 7. Related to Figure 7. (a) List of genes involved in regulating ferroptosis with reported positive (promoting) roles or negative (inhibiting) roles.

a

Cellular Functions	*Z*-score	*P*-value
Recruitment of neutrophils	3.23	4.05E-29
Cell movement of neutrophils	3.60	3.43E-27
Accumulation of neutrophils	2.75	1.64E-16
Degranulation of neutrophils	2.63	5.37E-17
Production of ROS	2.87	2.64E-20
Metabolism of ROS	2.57	1.67E-26
Biosynthesis of PUFAs	2.26	1.05E-17
Advanced malignant solid tumor	2.94	2.45E-38

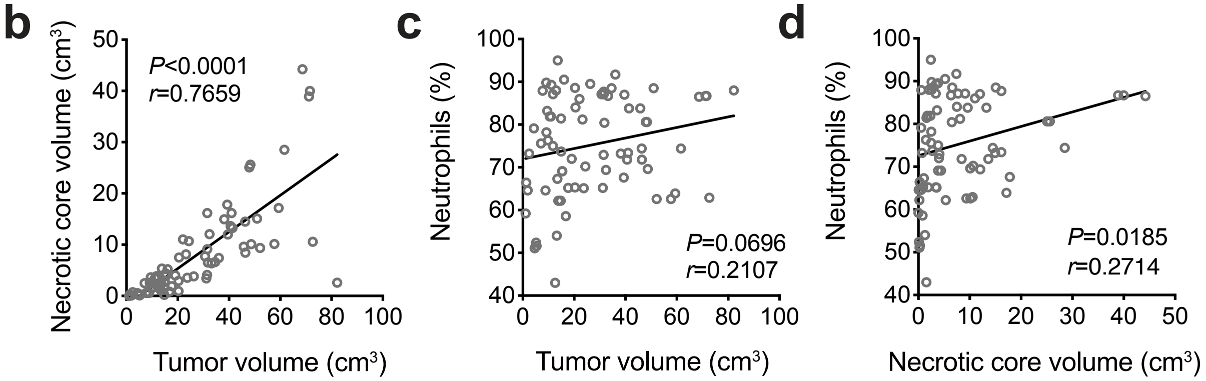

Supplementary Figure 8. Related to Figure 8. (a) Ingenuity pathway analysis (IPA) comparing differential gene expression in cellular tumor (CT) and tumor peri-necrotic zones (PNZ) and their corresponding cellular functions obtained from Ivy GBM Atlas. Genes with cellular functions listed here are those differentially upregulated in the tumor PNZ when compared to CT. Right-tailed Fisher's exact test. **(b)** Correlation analyses of pre-operative MRI-identified absolute tumor and necrosis volumes. Pearson's correlation coefficient test. **(c), (d)** Correlation analyses of pre-operative MRI-identified absolute tumor (c) and necrosis (d) volumes and peripheral neutrophil count reported in blood differentials in the GBM patient study cohort in Figure 8b. Pearson's correlation coefficient test; *P*-value is shown for each comparison. (b-d) n = 75, where n indicates total number of independently examined human subjects. Source data are provided as a Source Data file.

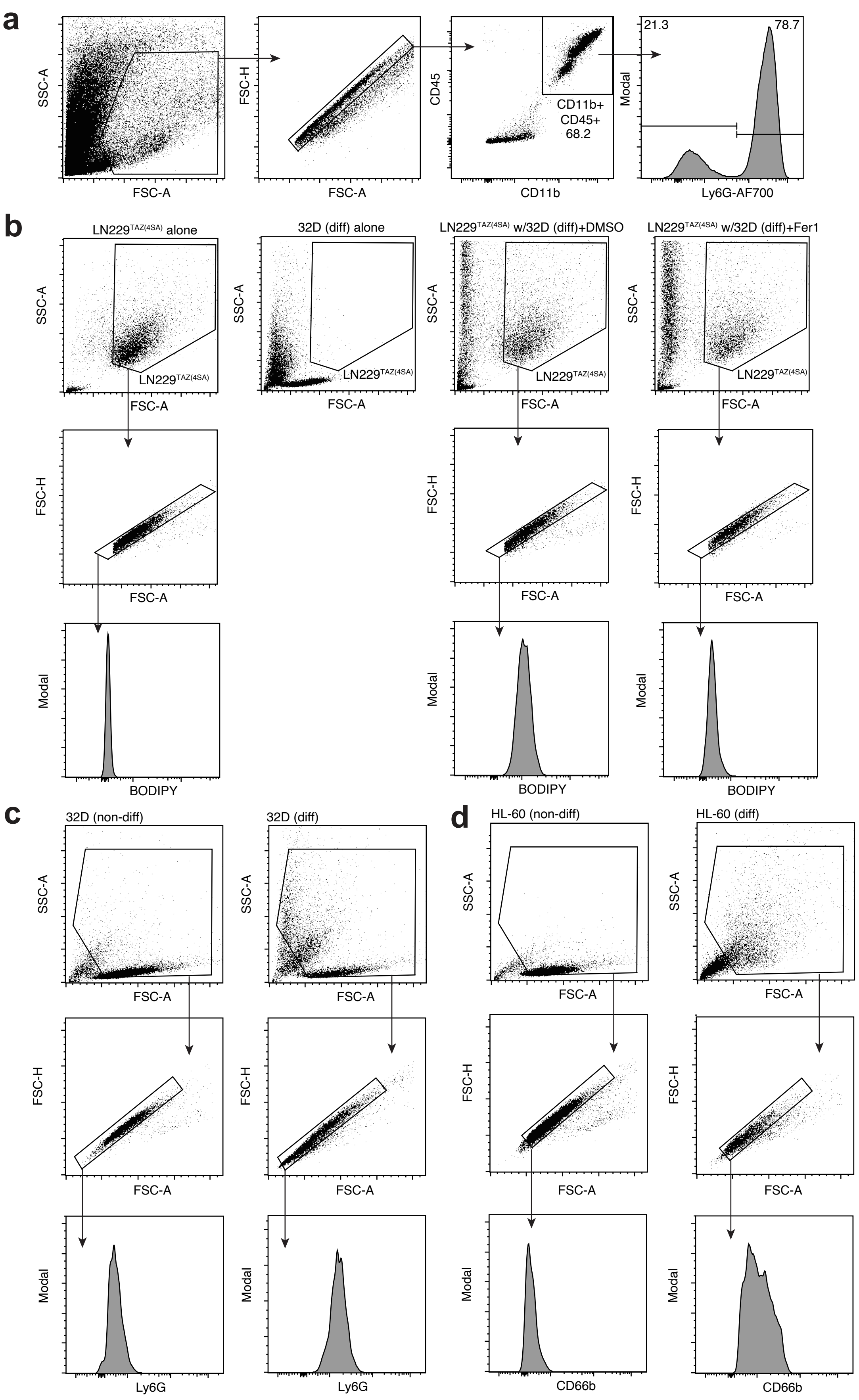

Supplementary Figure 9. Flow cytometry sequential gating/sorting strategies. (a) Gating strategies used for Figures 2c, 2e, and Supplementary Figure 2a. **(b)** Gating strategies used for Figure 4c and Supplementary Figure 4h. **(c)** Gating strategies used for Supplementary Figure 3i. **(d)** Gating strategies used for Supplementary Figure 3l.

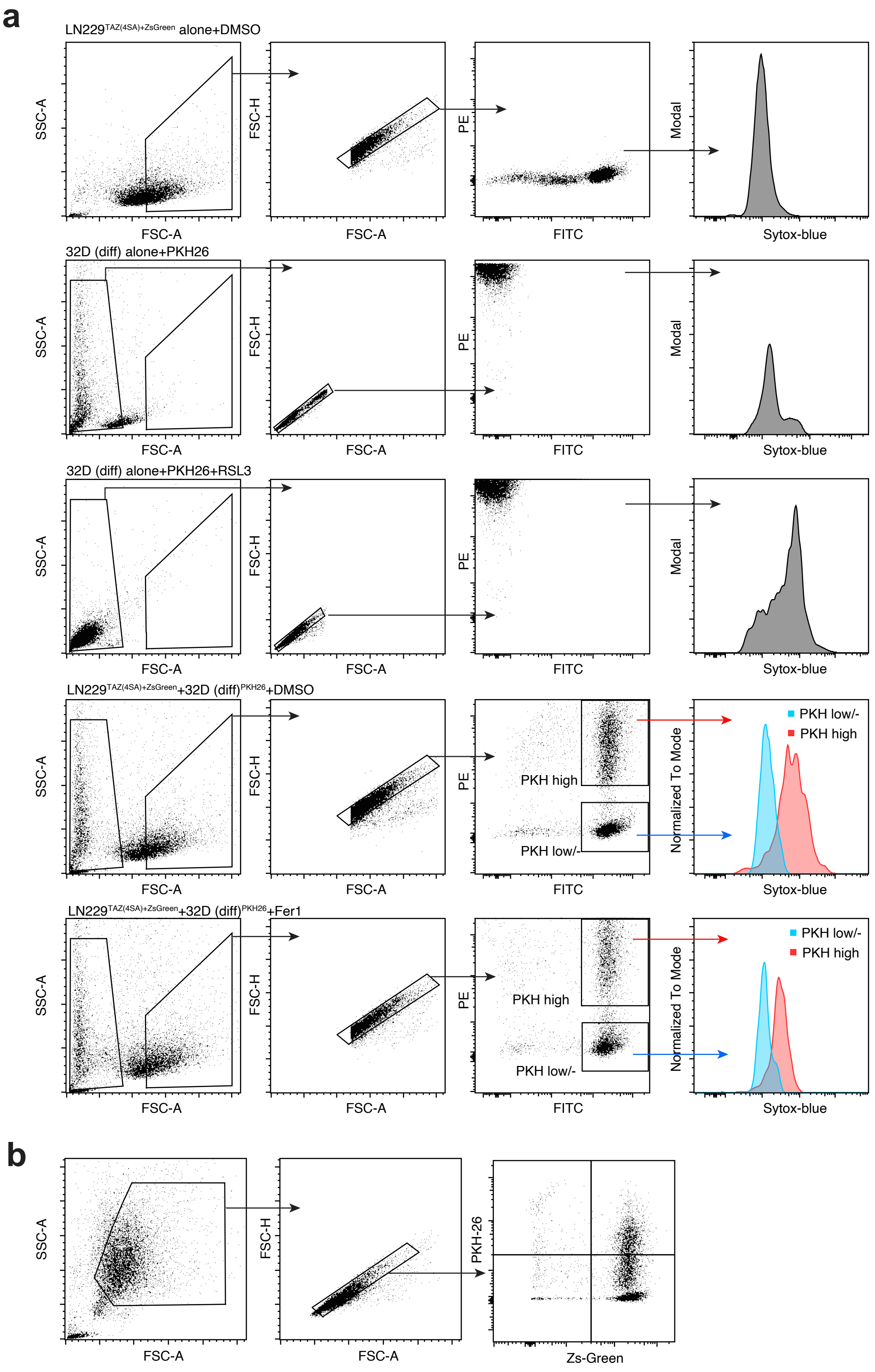

Supplementary Figure 10. Flow cytometry sequential gating/sorting strategies. (a) Gating strategies used for Figure 5i and Supplementary Figure 5e. **(b)** Gating strategies used for sorting in Supplementary Figure 5d.

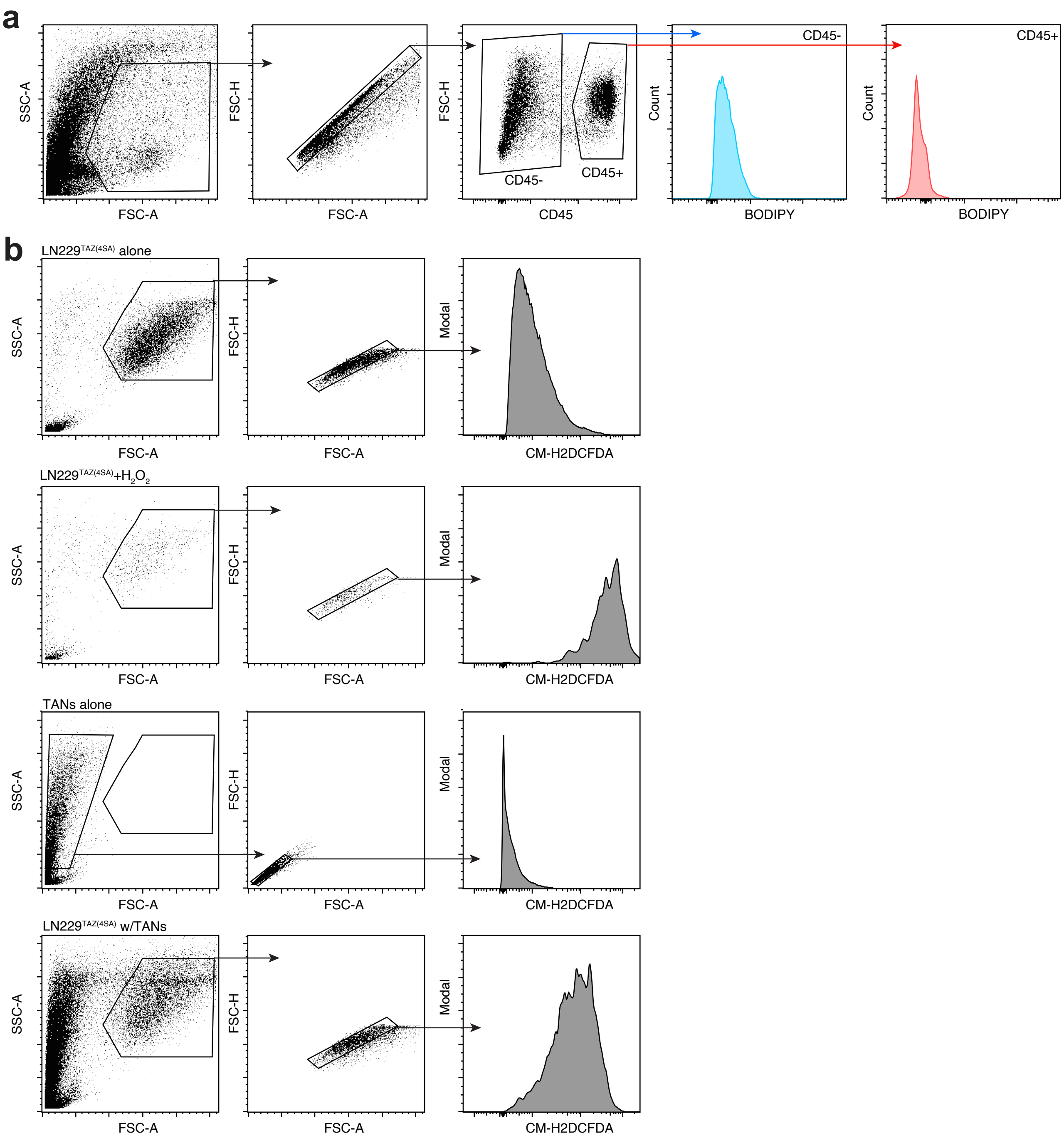

Supplementary Figure 11. Flow cytometry sequential gating/sorting strategies. (a) Gating strategies used for Figure 4h, Supplementary Figure 4i, and Supplementary Figure 4j. **(b)** Gating strategies used for Supplementary Figure 4e.

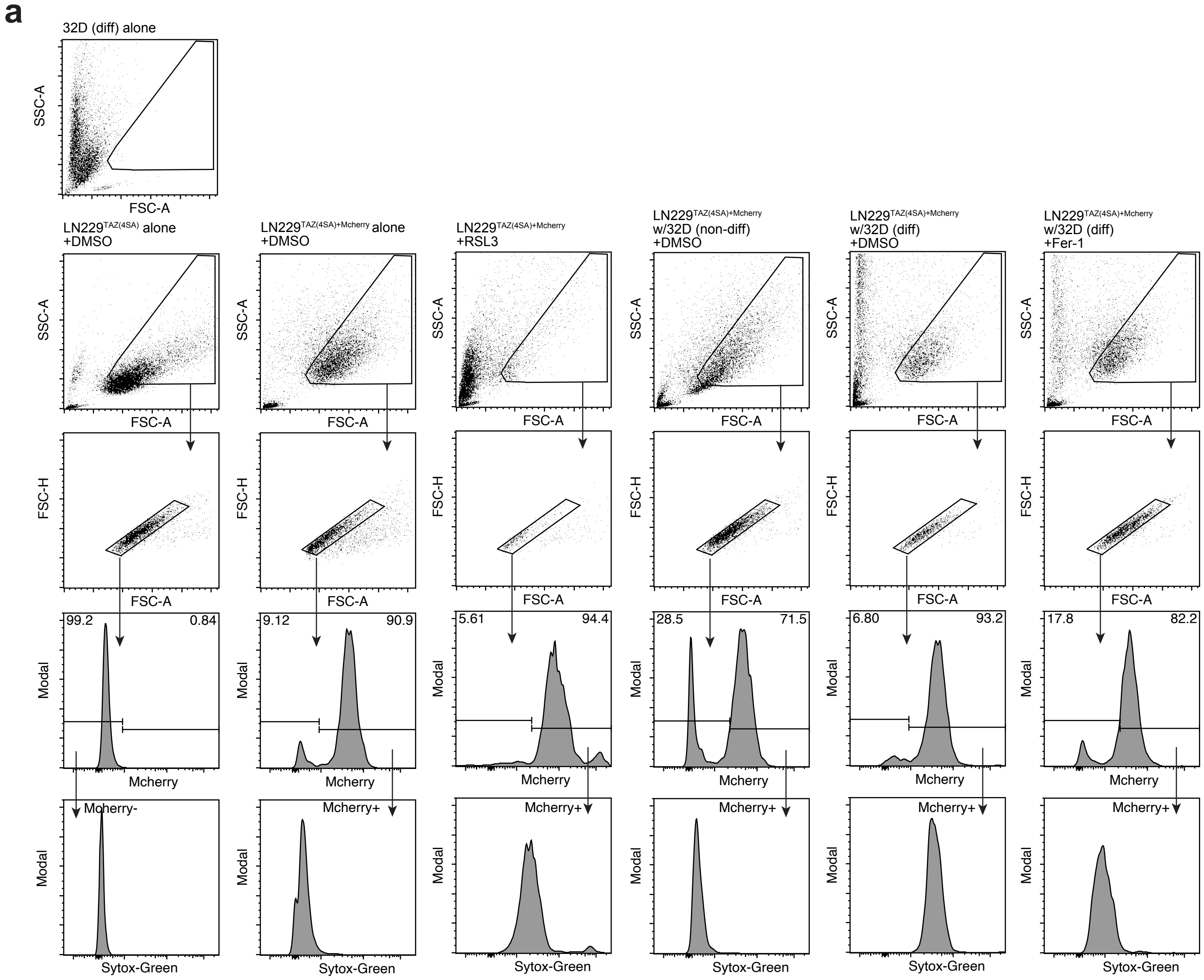

Supplementary Figure 12. Flow cytometry sequential gating/sorting strategies. (a) Gating strategies used for Supplementary Figure 3h and Supplementary Figure 4d.

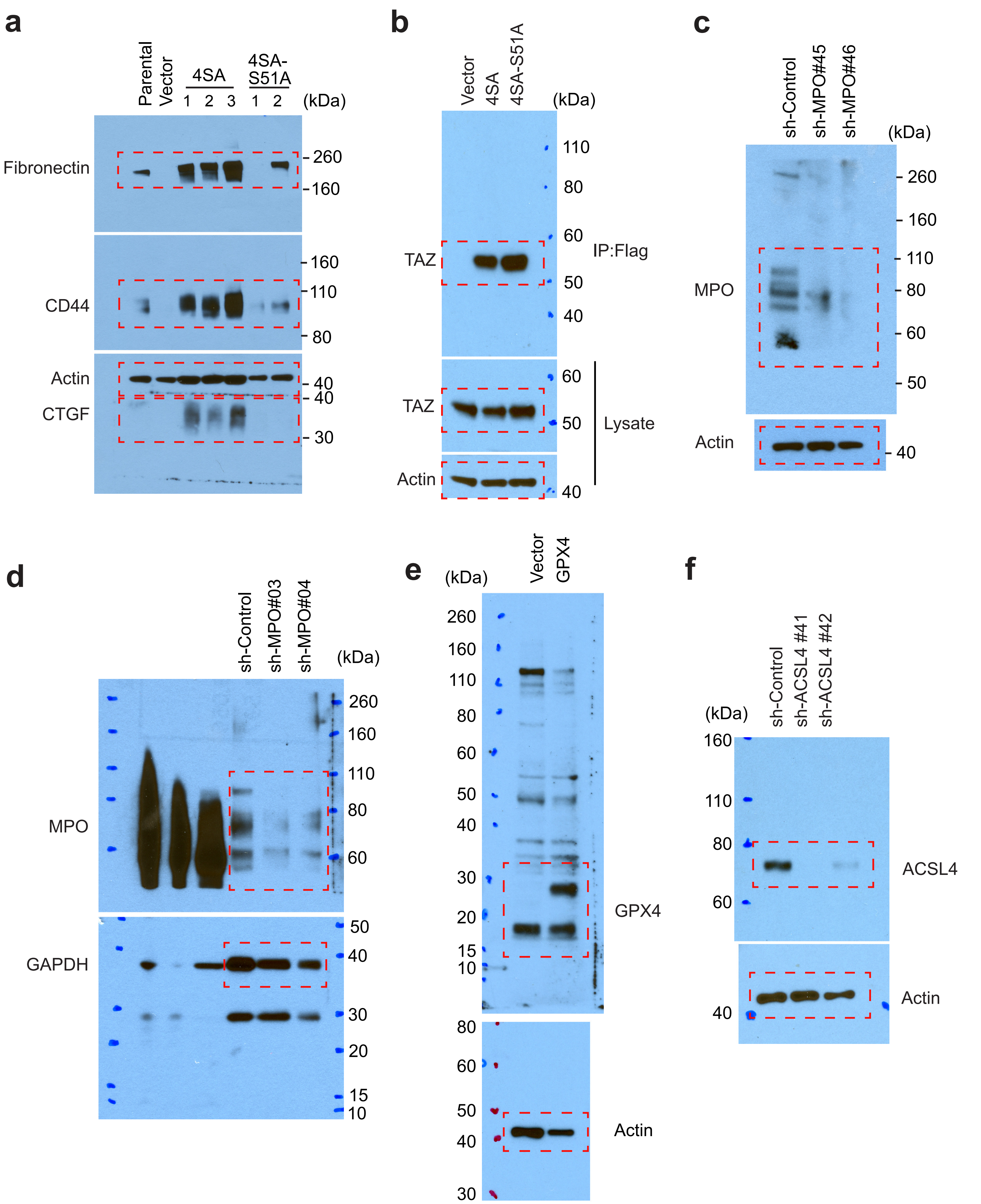

Supplementary Figure 13. Uncropped Blots. **(a)** For Figure 1c. **(b)** For Supplementary Figure 1a. **(c)** For Supplementary Figure 5h. **(d)** For Supplementary Figure 5i. **(e)** For Supplementary Figure 6a. **(f)** Supplementary Figure 6g.
