# Supplementary material for: Neutrophil-induced ferroptosis promotes tumor necrosis in glioblastoma progression
Source: Nat Commun. 2020 Oct 27;11:5424. doi: 10.1038/s41467-020-19193-y (PMC7591536; doi:10.1038/s41467-020-19193-y)
Supplement: Supplementary file 3 — Reporting Summary [file 41467_2020_19193_MOESM3_ESM.pdf]

nature research

Corresponding author(s): Wei Li

Last updated by author(s): 2020-9-22

Reporting Summary

Nature Research wishes to improve the reproducibility of the work that we publish. This form provides structure for consistency and transparency in reporting. For further information on Nature Research policies, see Authors & Referees and the Editorial Policy Checklist .

Statistics

For all statistical analyses, confirm that the following items are present in the figure legend, table legend, main text, or Methods section.

n/a	Confirmed	
☐	☒	The exact sample size (*n*) for each experimental group/condition, given as a discrete number and unit of measurement
☐	☒	A statement on whether measurements were taken from distinct samples or whether the same sample was measured repeatedly
☐	☒	The statistical test(s) used AND whether they are one- or two-sided<br>*Only common tests should be described solely by name; describe more complex techniques in the Methods section.*
☐	☒	A description of all covariates tested
☐	☒	A description of any assumptions or corrections, such as tests of normality and adjustment for multiple comparisons
☐	☒	A full description of the statistical parameters including central tendency (e.g. means) or other basic estimates (e.g. regression coefficient) AND variation (e.g. standard deviation) or associated estimates of uncertainty (e.g. confidence intervals)
☐	☒	For null hypothesis testing, the test statistic (e.g. *F*, *t*, *r*) with confidence intervals, effect sizes, degrees of freedom and *P* value noted<br>*Give P values as exact values whenever suitable.*
☒	☐	For Bayesian analysis, information on the choice of priors and Markov chain Monte Carlo settings
☒	☐	For hierarchical and complex designs, identification of the appropriate level for tests and full reporting of outcomes
☐	☒	Estimates of effect sizes (e.g. Cohen's *d*, Pearson's *r*), indicating how they were calculated

Our web collection on statistics for biologists contains articles on many of the points above.

Software and code

Policy information about availability of computer code

Data collection	N/A.
Data analysis	ImageJ 1.50, GraphPad Prism 8, Philips IntelliSpace PACS Enterprise 4.4, and FlowJo v10.

For manuscripts utilizing custom algorithms or software that are central to the research but not yet described in published literature, software must be made available to editors/reviewers. We strongly encourage code deposition in a community repository (e.g. GitHub). See the Nature Research guidelines for submitting code & software for further information.

Data

Policy information about availability of data

All manuscripts must include a data availability statement. This statement should provide the following information, where applicable:

- Accession codes, unique identifiers, or web links for publicly available datasets
- A list of figures that have associated raw data
- A description of any restrictions on data availability

The datasets used in this manuscript were downloaded from the Ivy Glioblastoma Atlas Project (https://glioblastoma.alleninstitute.org/) or cBioPortal (https://www.cbioportal.org/). Full scanned images of our immunoblotting films are provided with this paper. Representative flow cytometry gating strategies for each flow cytometry and sorting experiment are provided in the supplementary information. Source data from this study are also included with this paper as a source data file.

Field-specific reporting

Please select the one below that is the best fit for your research. If you are not sure, read the appropriate sections before making your selection.

Life sciences study design

All studies must disclose on these points even when the disclosure is negative.

Sample size	No sample size calculation was performed. Samples sizes were chosen based on if the differences between groups are biologically meaningful and are statistically significant. The Methods section and Legends contain a description of the sample size.
Data exclusions	No data points were excluded in our study.
Replication	Numbers of the experimental replication or the experiments that were performed independently for each specific result were indicated in the Methods section and Figure Legends.
Randomization	For cell experiments, all cells in each experiment were from the same pool of parental cells. All mice used in this study have the same genetic background (nude mice). Animals were from the same cohort. Animals were randomized in a blinded fashion when tumor cells from different groups were implanted. All animals were maintained in the same environment and handled by the same procedure.
Blinding	For data collected by objective instruments, such as plate readers, qPCR cyclers, microscopy software, flow cytometers, animal IVIS systems, and western blotting, the investigators were not blinded to group allocation during data collection. However, investigator bias is not considered to contribute to the data. For animal studies, the investigators were not blinded to group allocation during data collection when using the above mentioned objective instruments, but they were blinded during data analyses. Additionally, for all animal studies, randomization occurred in a blinded fashion. For clinical data analyses, investigators were not blinded during group allocation but were blinded during data analyses. Collected data were then verified by trained physicians who were blinded to group allocation.

Reporting for specific materials, systems and methods

We require information from authors about some types of materials, experimental systems and methods used in many studies. Here, indicate whether each material, system or method listed is relevant to your study. If you are not sure if a list item applies to your research, read the appropriate section before selecting a response.

Materials & experimental systems

n/a	Involved in the study	
☐	☒	Antibodies
☐	☒	Eukaryotic cell lines
☒	☐	Palaeontology
☐	☒	Animals and other organisms
☐	☒	Human research participants
☒	☐	Clinical data

Methods

n/a	Involved in the study	
☒	☐	ChIP-seq
☐	☒	Flow cytometry
☐	☒	MRI-based neuroimaging

Antibodies

Antibodies used	The information about the antibodies used in this study is described in the Methods.<br>Antibodies for flow cytometry: FITC anti-mouse CD11b (cat#101205, lot#B260639, Biolegend), PE anti-mouse CD45 (cat#103105, lot#B259877, Biolegend), BV421 Rat anti-mouse CD45 (cat#563890, lot#9170854, BD Biosciences), anti-mouse CD16/32 Fc receptor block (cat#553141, lot#9060742, BD Biosciences). Antibodies for IHC, ICC, and chromogenic immunodetection: Ultra-LEAF purified rat anti-mouse Ly-6G (1A8, cat#127620, lot#B203654 ,Biolegend), rabbit anti-mouse/human myeloperoxidase (cat#ab9535, lot# GR2459902-2 Abcam), anti-mouse/human PTGS2 (clone H-3, sc-376861, Santa Cruz), anti-mouse/human Cox2 (D5H5) XP (cat#12282S, lot#5, Cell Signaling Technologies), mouse anti-human CEACAM8/CD66b (cat#G10F5, lot#4, Novus Biologicals), rabbit anti-glutathione peroxidase 4, GPX4 (cat#ab125066, lot#GR3229900-5, Abcam), mouse-anti-HA.11 (16B12, MMS-101R, Biolegend), N-cadherin (13-A9, sc-59987, Santa Cruz Biotechnology), anti-mouse CD31 (89C2, 3528S, Cell Signaling Technologies), rabbit-anti-human/mouse GFAP (D1F4Q, 12389S, Cell Signaling Technologies), and rabbit-anti-mouse TMEM119 (28-3, ab209064, Abcam). Antibodies for immunoblotting: Mouse anti-human fibronectin (clone EP5, cat#sc-8422 lot#H3274, Santa Cruz), rabbit anti-human CD44 (cat#ab157107, lot#GR2139152-3, Abcam), rabbit anti-human CTGF (cat#86641, lot#1, Cell Signaling Technologies), mouse anti-β-Actin (cat#3700, lot#14, Cell Signaling Technologies), anti-mouse/human GPX4 (clone E-12, lot#H2719, cat#sc-166570, Santa Cruz), TAZ (V386, 4883S, Cell Signaling Technologies), mouse anti-human ACSL4 (F-4, sc-365230, Santa Cruz Biotechnology), anti-mouse/human MPO (ab9535, Abcam), anti-human MPO (E1E7I, 14569, Cell Signaling Technologies), anti-human GAPDH (0411, sc-47724, Santa Cruz Biotechnology), goat anti-rabbit HRP-conjugated antibody (7074S, Cell Signaling Technologies), and goat anti-mouse HRP-conjugated antibody (7076S, Cell Signaling Technologies). The human cytokine antibody array kit was purchased from Abcam (ab133998). The antibodies used for in vivo neutrophil depletion: InVivoPlus rat anti-mouse Ly6G, clone 1A8 (BE0075-1, BioXCell) and InVivoPlus rat IgG2a isotype control, clone 2A3 (BE0089, BioXCell).
Validation	The following antibodies were quality-checked and validated based on the information provided on the manufacturers' websites:<br>-FITC anti-mouse CD11b: https://www.biolegend.com/en-us/products/fitc-anti-mouse-human-cd11b-antibody-347<br>-PE anti-mouse CD45: https://www.biolegend.com/en-us/products/pe-anti-mouse-cd45-antibody-100<br>-BV421 Rat anti-mouse CD45: https://www.bdbiosciences.com/us/reagents/research/antibodies-buffers/immunology-

reagents/anti-mouse-antibodies/cell-surface-antigens/bv421-rat-anti-mouse-cd45-30-f11/p/563890
-Anti-mouse CD16/32 Fc receptor block: https://www.bdbiosciences.com/us/applications/research/b-cell-research/surface-markers/mouse/purified-rat-anti-mouse-cd16cd32-mouse-bd-fc-block-24g2/p/553142
-Ultra-LEAF purified rat anti-mouse Ly-6G: https://www.biolegend.com/en-us/products/leaf-low-endotoxin--azide-freepurified-anti-mouse-ly-6g-antibody-6357
-Rabbit anti-mouse/human myeloperoxidase: https://www.abcam.com/myeloperoxidase-antibody-ab9535.html
-Anti-mouse/human Cox2 (D5H5) XP: https://www.cellsignal.com/products/primary-antibodies/cox2-d5h5-xp-rabbit-mab/12282?site-search-type=Products&N=4294956287&Ntt=12282s&fromPage=plp&_requestid=3827680
-Mouse anti-human CEACAM8/CD66b: https://www.novusbio.com/products/ceacam8-cd66b-antibody-g10f5_nb100-77808
-Rabbit anti-glutathione peroxidase 4, GPX4 : https://www.abcam.com/glutathione-peroxidase-4-antibody-epncir144-ab125066.html
-Mouse-anti-HA.11: https://www.biolegend.com/en-us/products/anti-ha-11-epitope-tag-antibody-11071
-Anti-human N cadherin: https://www.scbt.com/p/n-cadherin-antibody-13a9
-Anti-mouse CD31 : https://www.cellsignal.com/products/primary-antibodies/cd31-pecam-1-89c2-mouse-mab/3528?Ntk=Products&site-search-type=Products&N=4294956287&Ntt=3528s&fromPage=plp&_requestid=1132486
-Rabbit-anti-human/mouse GFAP: https://www.cellsignal.com/products/primary-antibodies/gfap-d1f4q-xp-rabbit-mab/12389?Ntk=Products&site-search-type=Products&N=4294956287&Ntt=12389s&fromPage=plp&_requestid=1132555
-Rabbit-anti-mouse TMEM119: https://www.abcam.com/tmem119-antibody-28-3-microglial-marker-ab209064.html
-Mouse anti-human fibronectin: https://www.scbt.com/p/fibronectin-antibody-ep5
-Rabbit anti-human CD44: https://www.abcam.com/cd44-antibody-ab157107.html
-Rabbit anti-human CTGF: https://www.cellsignal.com/products/primary-antibodies/ctgf-d8z8u-rabbit-mab/86641
-Mouse anti-β-Actin: https://www.cellsignal.com/products/primary-antibodies/b-actin-8h10d10-mouse-mab/3700
-Anti-mouse/human GPX4: https://www.scbt.com/p/gpx-4-antibody-e-12
-TAZ (V386): https://www.cellsignal.com/products/primary-antibodies/taz-v386-antibody/4883?Ntk=Products&site-search-type=Products&N=4294956287&Ntt=4883s&fromPage=plp&_requestid=1132675
-Mouse anti-human ACSL4: https://www.scbt.com/p/acsl4-antibody-f-4?requestFrom=search
-Anti-mouse/human MPO: https://www.abcam.com/myeloperoxidase-antibody-ab9535.html
-Anti-human MPO: https://www.cellsignal.com/products/primary-antibodies/myeloperoxidase-e1e7i-xp-rabbit-mab/14569?Ntk=Products&Ntt=14569
-Anti-human GAPDH: https://www.scbt.com/p/gapdh-antibody-0411?requestFrom=search
-Goat anti-rabbit HRP-conjugated antibody: https://www.cellsignal.com/products/wb-ip-reagents/phototope-hrp-western-blot-detection-system-anti-rabbit-igg-hrp-linked-antibody/7074.html?bvstate=pg:2/ct:r
-Goat anti-mouse HRP-conjugated antibody: https://www.cellsignal.com/products/secondary-antibodies/anti-mouse-igg-hrp-linked-antibody/7076?Ntk=Products&site-search-type=Products&N=4294956287&Ntt=goat+anti-mouse+hrp-conjugated+antibody&fromPage=plp
-InVivoPlus rat anti-mouse Ly6G, clone 1A8: https://bxcell.com/product/invivomab-anti-m-ly-6g/
-InVivoPlus rat IgG2a isotype control: https://bxcell.com/product/rat-igg2a-isotype-control/
-Anti-human cytokine antibody array kit: https://www.abcam.com/cytokine-array-human-cytokine-antibody-array-membrane-80-targets-ab133998.html

Eukaryotic cell lines

Policy information about cell lines

Cell line source(s)	Human GBM cell lines: LN229 (CRL-2611), U87 MG (HTB-14), LN18 (CRL-2610), murine myeloblastic cell line, 32D Clone 3 (CR-11346), and human promyelocytic leukemia cell line, HL-60 (CCL-240), were purchased from ATCC.
Authentication	None of the cell lines used were authenticated.
Mycoplasma contamination	All cell lines were tested negative for mycoplasma contamination.
Commonly misidentified lines (See ICLAC register)	None.

Animals and other organisms

Policy information about studies involving animals; ARRIVE guidelines recommended for reporting animal research

Laboratory animals	Six-to-eight-week-old female athymic nude mice (Nu(NCr)-Foxn1nu Strain Code: 490, Charles River) were used for the GBM xenograft mouse models. All animals were housed in a room with a 12-h light/dark cycle with free access to a standard rodent diet and water at ambient temperature maintained between 18-23 degrees Celsius and humidity between 40-60%.
Wild animals	This study did not involve wild animals.
Field-collected samples	This study did not involve samples collected from the field.
Ethics oversight	All animal experiments described in this study were carried out with the approval of the Penn State University Institutional Animal Care and Use Committee and in accordance with its guidelines.

October 2018

Note that full information on the approval of the study protocol must also be provided in the manuscript.

Human research participants

Policy information about studies involving human research participants

Population characteristics	Specimens and study subjects included in Figures 8c, 8d, and 8e were obtained from a cohort of deceased patients diagnosed with glioblastoma at Penn State Milton S. Hershey Medical Center. This project was therefore not considered human subject research by the Penn State Institutional Review Board (IRB). Human subjects included in Figures 8b, S8b, S8c, and S8d are patients who were older than 18 and diagnosed with glioblastoma at the Penn State Neurooncology clinic between 2018 and 2019. Both genders were included in this study. In addition, for this MRI study, only pre-existing data were obtained via review of electronic medical records (EMRs) as well as imaging studies (MRIs). No further data collection was performed.
Recruitment	No recruitment was needed for our study.
Ethics oversight	All human subject research included in this manuscript has been reviewed and approved by the Penn State Institutional Review Board (IRB). Per Penn State IRB, all human subject research included in this manuscript was exempt from informed consent requirements.

Note that full information on the approval of the study protocol must also be provided in the manuscript.

Flow Cytometry

Plots

Confirm that:

☐ The axis labels state the marker and fluorochrome used (e.g. CD4-FITC).

☒ The axis scales are clearly visible. Include numbers along axes only for bottom left plot of group (a 'group' is an analysis of identical markers).

☐ All plots are contour plots with outliers or pseudocolor plots.

☒ A numerical value for number of cells or percentage (with statistics) is provided.

Methodology

Sample preparation	Following single cell dissociation, singly-suspended live cells were first blocked with purified rat anti-mouse CD16/32 Fc receptor block (553141, BD Biosciences) prior to antibody labeling. After that, single-cell suspensions at a concentration of 1×10^6 cells/ml prepared from fresh tumor tissue were labeled by surface markers via anti-CD45 (clone 30-F11), anti-CD11b (clone M1/70), anti-Ly6G (clone 1A8) antibodies at room temperature for 20 minutes (protected from lights). Cells were then washed three times, resuspended in 1 ml of DPBS, and run on an LSRFortessa (BD Biosciences) cell analyzer in the Penn State College of Medicine's Flow Cytometry Core.
Instrument	16-color LSRFortessa (BD Biosciences) for flow cytometry; 17-color BD FACS Aria SORP high-performance cell sorter (BD Biosciences) for cell sorting
Software	FlowJo™ v10
Cell population abundance	For sorting: 1-2 million GFP+ tumor cells were obtained post-sorting. Purity of sorted cells were verified via fluorescence microscopy.
Gating strategy	Relevant gating strategies provided in the supplementary. Briefly, cells were first gated by FSC and SSC, followed by FSC-H and FSC-A for single cell populations, followed by positive and negative population with respective fluorochrome.

☒ Tick this box to confirm that a figure exemplifying the gating strategy is provided in the Supplementary Information.

Magnetic resonance imaging

Experimental design

Design type	Imaging analyses were conducted retrospectively. All brain MRI scans from the patient cohort were ordered as part of their routine pre-operative procedures. T1 weighted FS MRI scans used for volumetric quantification of tumor and necrosis areas were obtained through the centralized radiology imaging system, Phillips PACS, at Penn State Hershey Medical Center. No modification or additional processing was performed on these MRI images obtained prior to volumetric quantification. Procedures of obtaining radiological imaging data were approved by the Institutional Review Board (IRB) of Penn State Hershey Medical Center.
Design specifications	Volumetric quantification of the tumor and necrosis areas were performed retrospectively using MRI images available on Phillips PACS at Penn State Hershey Medical Center. Images were obtained and quantified without any additional modification or processing.
Behavioral performance measures	No behavioral performance was measured for the volumetric quantification of the tumor and necrosis areas.

Acquisition

Imaging type(s)	Pre-surgical, post-contrast axial T1-weighted fat suppression (T1 FS) MRI images
Field strength	3 Tesla
Sequence & imaging parameters	T1-weighted fat suppression (T1 FS) MRI; field of view: axial; sequence thickness 5-6 mm/slice
Area of acquisition	whole brain scans were used.

Diffusion MRI ☐ Used ☒ Not used

Preprocessing

Preprocessing software	Phillips PACS
Normalization	No normalization was used for volumetric quantification of the tumor and necrosis areas.
Normalization template	No normalization template was used for the volumetric quantification of the tumor and necrosis areas.
Noise and artifact removal	No noise or artifact removal was performed for the volumetric quantification.
Volume censoring	Volumetric measurements were obtained using Phillips PACS.

Statistical modeling & inference

Model type and settings	No statistical modeling or interference was used for the volumetric quantification of the tumor and necrosis areas.
Effect(s) tested	No specific effect was tested for the volumetric quantification of the tumor and necrosis areas.

Specify type of analysis: ☐ Whole brain ☒ ROI-based ☐ Both

Anatomical location(s): Whole tumors and necrotic regions within tumors.

Statistic type for inference (See Eklund et al. 2016)	No statistic type for inference was used for the volumetric quantification of the tumor and necrosis areas.
Correction	No correction was performed for the volumetric quantification of the tumor and necrosis areas.

Models & analysis

n/a	Involved in the study
☒	☐ Functional and/or effective connectivity
☒	☐ Graph analysis
☒	☐ Multivariate modeling or predictive analysis
